# Cytosolic phospholipase A_2_-α participates in lipid body formation and PGE_2_ release in human neutrophils stimulated with an l-amino acid oxidase from *Calloselasma rhodostoma* venom

**DOI:** 10.1038/s41598-020-67345-3

**Published:** 2020-07-03

**Authors:** Mauro Valentino Paloschi, Jéssica Amaral Lopes, Charles Nunes Boeno, Milena Daniela Souza Silva, Jaína Rodrigues Evangelista, Adriana Silva Pontes, Sulamita da Silva Setúbal, Cristina Matiele Alves Rego, Neriane Monteiro Néry, Alex Augusto Ferreira e Ferreira, Weverson Luciano Pires, Kátia Paula Felipin, Gabriel Eduardo Melim Ferreira, Patrícia Torres Bozza, Juliana Pavan Zuliani

**Affiliations:** 1 Laboratório de Imunologia Celular Aplicada à Saúde, Fundação Oswaldo Cruz, FIOCRUZ Rondônia, Porto Velho, RO Brazil; 2 Laboratório de Epidemiologia Genética (EpiGen), Fundação Oswaldo Cruz, FIOCRUZ Rondônia, Porto Velho, RO Brazil; 3 Laboratório de Imunofarmacologia, Instituto Oswaldo Cruz, IOC, Rio de Janeiro, RJ Brazil; 4 Centro de Estudos de Biomoléculas Aplicadas à Saúde (CEBio), Fundação Oswaldo Cruz, FIOCRUZ Rondônia e Departamento de Medicina, Universidade Federal de Rondônia, UNIR, Porto Velho, RO Brazil; 5 Programa de Pós-Graduação em Biologia Experimental, Departamento de Medicina, Universidade Federal de Rondônia, UNIR, Porto Velho, RO Brazil

**Keywords:** Inflammation, Innate immune cells

## Abstract

Cr-LAAO, an l-amino acid oxidase isolated from *Calloselasma rhodosthoma* snake venom, has been demonstrated as a potent stimulus for neutrophil activation and inflammatory mediator production. However, the mechanisms involved in Cr-LAAO induced neutrophil activation has not been well characterized. Here we investigated the mechanisms involved in Cr-LAAO-induced lipid body (also known as lipid droplet) biogenesis and eicosanoid formation in human neutrophils. Using microarray analysis, we show for the first time that Cr-LAAO plays a role in the up-regulation of the expression of genes involved in lipid signalling and metabolism. Those include different members of phospholipase A_2,_ mostly cytosolic phospholipase A_2_-α (cPLA_2_-α); and enzymes involved in prostaglandin synthesis including cyclooxygenases 2 (COX-2), and prostaglandin E synthase (PTGES). In addition, genes involved in lipid droplet formation, including perilipin 2 and 3 (PLIN 2 and 3) and diacylglycerol acyltransferase 1 (DGAT1), were also upregulated. Furthermore, increased phosphorylation of cPLA_2_-α, lipid droplet biogenesis and PGE_2_ synthesis were observed in human neutrophils stimulated with Cr-LAAO. Treatment with cPLA_2_-α inhibitor (CAY10650) or DGAT-1 inhibitor (A922500) suppressed lipid droplets formation and PGE_2_ secretion. In conclusion, we demonstrate for the first time the effects of Cr-LAAO to regulate neutrophil lipid metabolism and signalling.

## Introduction

Neutrophils are the first leukocytes to migrate to the inflammatory sites in response to chemotactic factors, where they phagocytose pathogens and release lipid mediators that regulate inflammation^1–3^. Among the lipid mediators, prostaglandin E_2_ (PGE_2_) acts on blood flow, oedema, and pain^4–6^. Prostaglandins are arachidonic acid metabolites that undergo sequential action by three important enzymes, phospholipase A_2_ (PLA_2_), and cyclooxygenases (COX-1 and COX-2) and terminal prostanoid synthase^7^.

There are several classes of PLA_2_ in mammals: secreted PLA_2_, cytosolic PLA_2_ (cPLA_2_), calcium-independent PLA_2_ (iPLA_2_), lysosomal PLA_2_, and platelet activation acetylhydrolase (PAF-AH), each of which plays a role in generating eicosanoids^8^. cPLA_2_ phosphorylation, especially the α-type cPLA_2_ (cPLA_2_-α), cleaves phospholipids, generating free fatty acids, such as arachidonic acid (AA), which is oxidized by COXs^9^. COXs are responsible for catalyzing the oxygenation and cyclization reactions of fatty acids to form prostaglandin G_2_ (PGG_2_). COXs also present a hydroperoxidase activity that converts PGG_2_ to PGH_2_, which is then converted to PGE_2_ by prostaglandin E synthase (PTGES)^9,10^.

Lipid bodies (LBs; also known as lipid droplets) are dynamic and well regulated organelles that have their formation up-regulated in activated leukocytes and play roles during inflammation^11,12^. LBs are composed of lipidic organic compounds and diverse functional proteins, such as perilipines (PLINs), which are activated in metabolism, cell signalling, and inflammation^12–16^. In addition to PLINs, diglyceride acyltransferases (DGATs) are central enzymes for the synthesis of thiacylglycerol from fatty acid substrates derived from lipogenesis^17^. Accumulating evidence have indicated that LBs act as platforms for the synthesis of inflammatory lipid mediators by compartmentalizing the substrate AA as well as a large number of proteins involved in eicosanoid synthesis, such as PLA_2_s, COX, PTGES, and 5- and 15-lipoxygenases (5-LO and 15-LO)^18–22^.

Cr-LAAO, an L-amino acid oxidase (LAAO, EC 1.4.3.2) isolated from *Calloselasma rhodosthoma* snake (Malaysia viper) venom are flavoenzymes present at relatively high concentrations in most snake venoms. This enzyme has pharmacological effects, including haemolysis and haemorrhage, in addition to the stimulation of apoptosis, inhibition or induction of platelet aggregation, oedema formation, and activation of leukocytes. It also plays an important role in bactericidal, cytotoxic, antiparasitic, antitumor, and antiviral activities^23–27^. Pontes et al.^28,29^ showed that Cr-LAAO activates isolated human neutrophils leading to ROS production (superoxide anion and hydrogen peroxide), stimulation of phagocytosis and chemotaxis by p38 mitogen-activated protein kinase (p38MAPK), and activation of phosphoinositide-3 kinase (PI3K). Moreover, Paloschi et al.^30^ reported that Cr-LAAO can activate the NADPH oxidase complex with PKCα participation. The toxin also induces the release of myeloperoxidase (MPO), cytokines (IL-8, IL-6, and TNF-α), neutrophil extracellular traps (NETs), and lipid mediators (LTB_4_ and PGE_2_)^28,29^.

The biogenesis of LBs during inflammation depends on the cell type and the stimulus that the cell undergoes, since it depends on specific signalling pathways^31^. Since Cr-LAAO is a molecule capable of stimulating the activation of neutrophils and production of eicosanoids, we hypothesized that Cr-LAAO regulates LBs formation in neutrophils. Here we provide evidence that Cr-LAAO has major effects on human neutrophil lipid metabolism and signaling.

## Results

### Microarray and gene analysis expression

**Figure 1:**
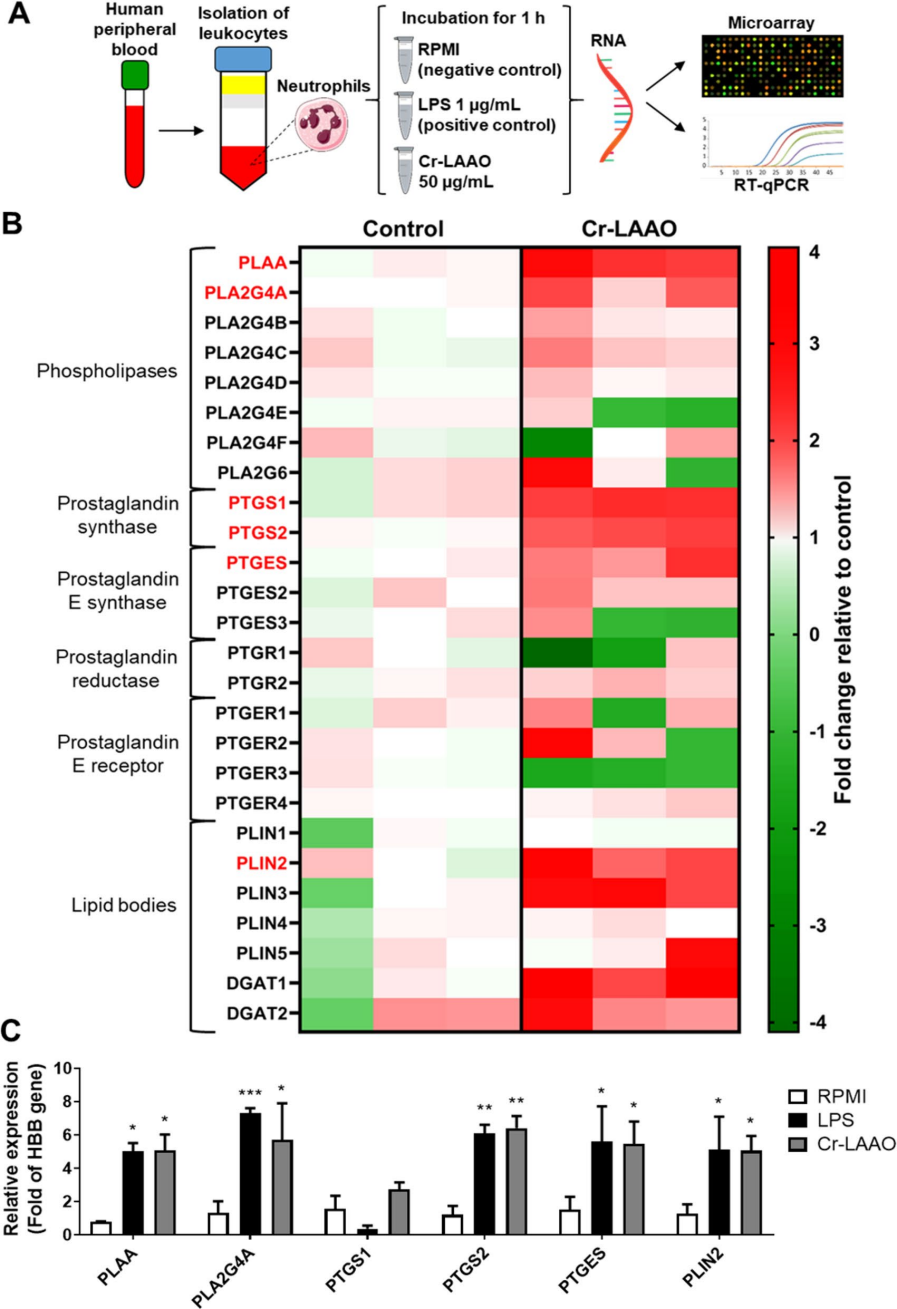
Gene expression of COX pathway. The microarray and qRT-PCR were performed with 2 × 10^5^ human neutrophils from 3 different donors, stimulated with Cr-LAAO (50 μg/mL), LPS (1 μg/mL; positive control) or RPMI (negative control) for 1 h at 37 °C and 5% CO_2_. This figure was created using images from Servier Medical Art Commons Attribution 3.0 Unported License (https://smart.servier.com) (**A**). Servier Medical Art by Servier is licensed under a Creative Commons Attribution 3.0 Unported License. The microarray fold change was represented in up regulation (red) and down regulation (green) for cells stimulated with Cr-LAAO in relation to control. The genes shown were for phospholipase A2-activating protein (PLAA), group IVA (PLA2G4A), IVB (PLA2G4B), IVC (PLA2G4C), IVD (PLA2G4D), IVE (PLA2G4E), IVF (PLA2G4F) and VI (PLA2G6) phospholipase A2, prostaglandin-endoperoxide synthase 1 (PTGS1) and 2 (PTGS2), prostaglandin E synthase 1 (PTGES), 2 (PTGES2) and 3 (PTGES3), prostaglandin reductase 1 (PTGR1) and 2 (PTGR2), prostaglandin E receptor 1 (PTGER1), 2 (PTGER2), 3 (PTGER3) and 4 (PTGER4), perilipin 1–5 (PLIN1, PLIN12, PLIN3, PLIN4 and PLIN5), diacylglycerol O-acyltransferase 1 and 2 (DGAT1 and DGAT2) (**B**). The genes analyzed by qRT-PCR were PLAA, PLA2G4A, PTGS1, PTGS2, PTGES and PLIN2. Hemoglobin subunit beta (HBB) was used as reference gene for normalization (**C**). Values are mean S.E.M. from 3 donors. *P < 0.05, **P < 0.01, ***P < 0.001, ****P < 0.0001 compared to negative control (Data were presented with ANOVA followed by Dunnett post-test).

Microarray-based gene expression analysis allows the detection of approximately 22,000 genes, among which genes related to the COX pathway and lipid body were selected. The data were expressed in a heatmap as up-regulation (red) when the expression was higher in Cr-LAAO-stimulated neutrophils versus the negative control, and as down-regulation (green) when the expression was higher in the negative control than in the stimulated neutrophils. PLA_2_s-expressed genes were divided into cytosolic and secreted forms. The cytosolic forms were selected for this study, while the secreted forms are presented in Table S1. Group IV PLA_2_ (PLA2G4) subtypes (A, B, C, D), and PLA_2_ activating protein (PLAA) were predominantly up-regulated in all samples stimulated with Cr-LAAO. The PLA2G4 E and F subtypes, as well as group VI PLA_2_ (PLA2G6) did not show a consistent expression in all samples. COX-1 (PTGS1), COX-2 (PTGS2), prostaglandin reductase 2 (PTGR2), prostaglandin E synthase 1 (PTGES) and 2 (PTGES2), and prostaglandin E receptor subtype EP4 (PTGER4) genes were also up-regulated in all samples stimulated with Cr-LAAO. Genes PTGES3, PTGR1, and PTGER1, 2, and 3 did not present equivalent expression in all samples analysed. Regarding genes involved in lipid body structure, we evaluated the expression of perilipines 1 to 5, but only PLIN2 and PLIN3 were up-regulated in all samples stimulated with Cr-LAAO. In addition, DGAT1 and DGAT2 enzymes that have major roles in LB formation were also up-regulated. To confirm the microarray gene expression profile, qRT-PCR was performed for PTGS1, PTGS2, PLAA, PTGES, PLA2G4A, and PLIN2 genes. The results showed a statistically significant increase in gene expression in the LPS- and Cr-LAAO-stimulated neutrophil samples compared to the negative control for all genes tested, except for PTGS1, concurring with the up-regulated genes observed in the microarray assay (Fig. 1).

### Cr-LAAO induces cPLA_2_-α and COX accumulation in human neutrophils

**Figure 2:**
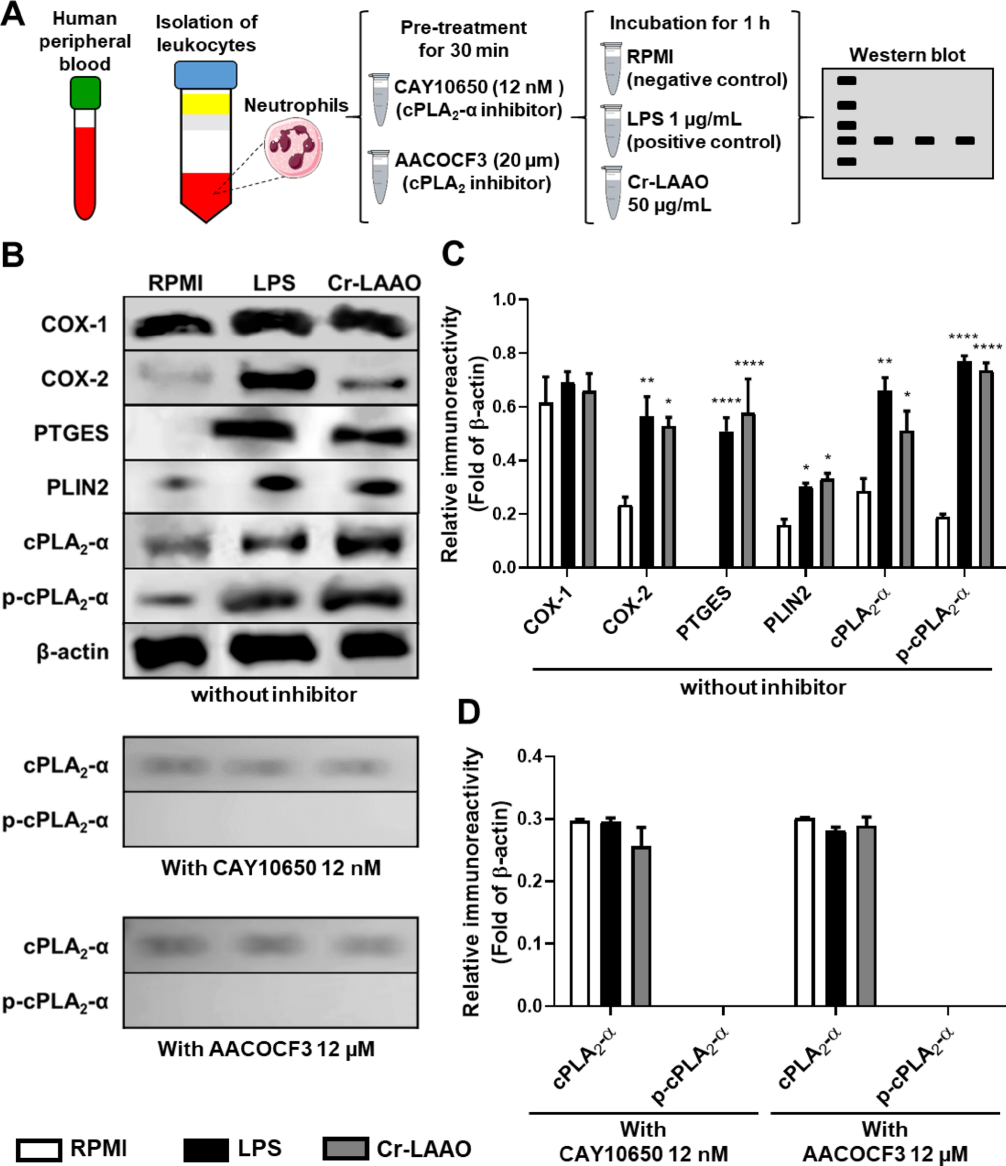
Protein expression of cPLA_2_-α and COX in neutrophils. Western blot of cPLA_2_-α, p-cPLA_2_-α_,_ COX-1, COX-2 and β-actin using human neutrophils (1 × 10^7^) stimulated Cr-LAAO (50 μg/mL), LPS (1 μg/mL; positive control) or RPMI (negative control) pre-treated or not with CAY10650 (cPLA_2_-α inhibitor, 12 nM for 30 min) or AACOCF3 (cPLA_2_ inhibitor, 20 µM for 30 min) for 1 h at 37 °C and 5% CO_2_. This figure was created using images from Servier Medical Art Commons Attribution 3.0 Unported License (https://smart.servier.com) (**A**). Servier Medical Art by Servier is licensed under a Creative Commons Attribution 3.0 Unported License. Figure representative of one experiment of three independent experiments (**B**). Relative immunoreactivity analysis (fold of β-actin) of the western blots from COX-1, COX-2, PTGES, PLIN2, cPLA_2_-α and p-cPLA_2_-α proteins without inhibitor (**C**); cPLA_2_-α and p-cPLA_2_-α proteins from neutrophils pre-treated with CAY10650 or AACOCF3 (**D**). Values are mean S.E.M. from 3 donors. *P < 0.05, **P < 0.01, ***P < 0.001, ****P < 0.0001 compared to negative control (Data were presented with ANOVA followed by Dunnett post-test).

Figure 2 shows the western blot analysis of isolated neutrophils incubated with RPMI (negative control), LPS (positive control), or Cr-LAAO (50 µg/mL) for 1 h. COX-1 expression was evidenced in all conditions analysed, including the negative control, proving to be constitutive. With respect to COX-2 expression, we observed a larger protein expression in samples containing LAAO and LPS when compared to RPMI, demonstrating COX-2 accumulation. Furthermore, the phosphorylated form of cPLA_2_ (p-cPLA_2_-α) has been shown to be expressed in cells stimulated with Cr-LAAO and LPS.

To confirm the action of the cPLA2-α inhibitor (CAY10650), protein expression analysis for cPLA_2_ and p-cPLA_2_-α was performed on neutrophils pretreated with 12 nM of CAY10650. Results showed that there was expression of cPLA2-α in the native form but there was no expression of the phosphorylated cPLA2-α (p-cPLA2-α) in cells pretreated with both concentrations of the inhibitor. In comparison, western blotting was also performed for cPLA2 and p-cPLA2-α in neutrophils pretreated with AACOCF3 (cPLA2-α inhibitor) at 20 µM. Results showed that as well as with CAY10650, it was observed cPLA2-α expression in the native form this was not observed the p-cPLA2-α expression in neutrophils pretreated with the inhibitor (Fig. 2) confirming that both CAY10650 and AACOCF3 inhibit cPLA2-α.

**Figure 3:**
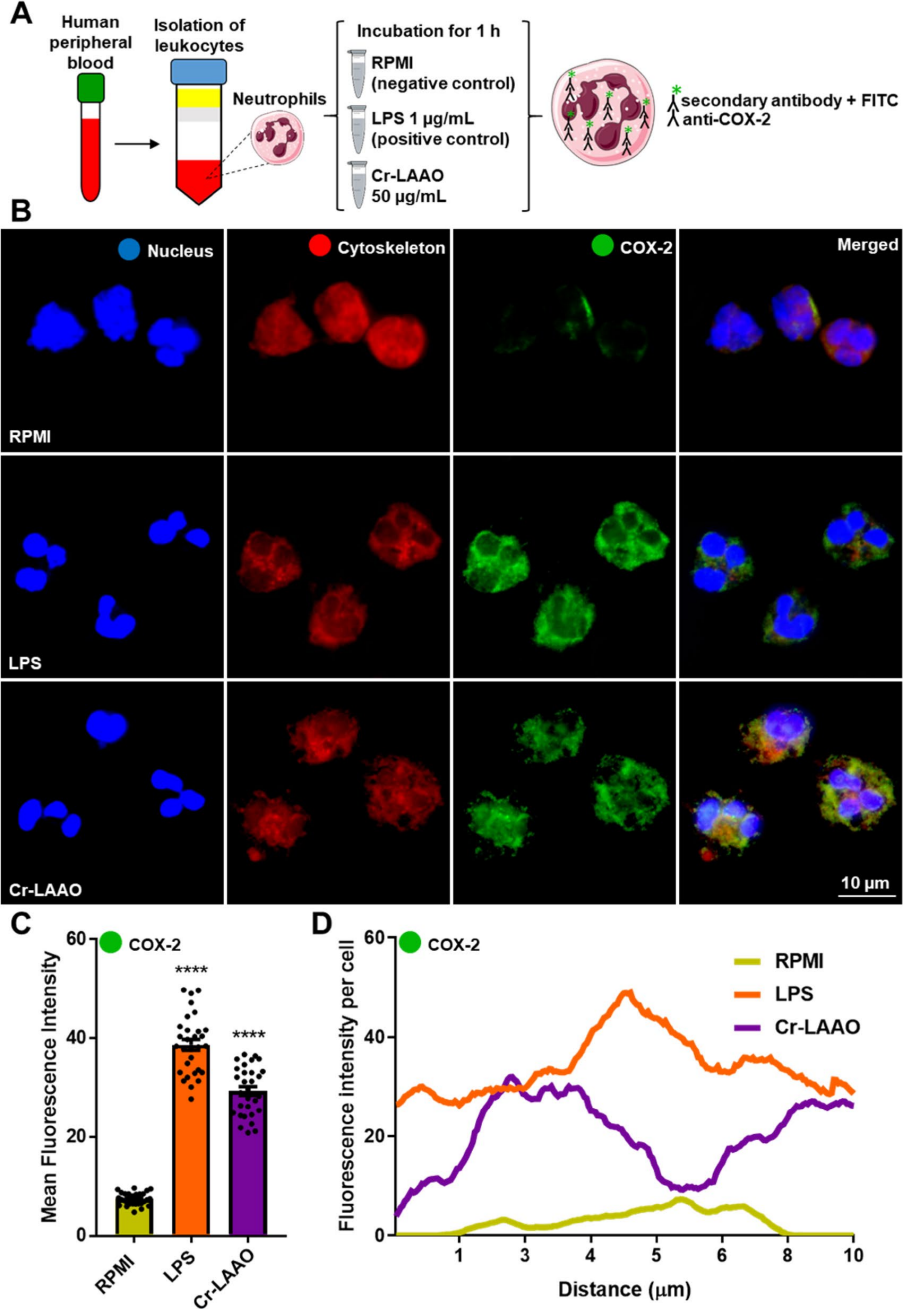
Immunofluorescence of COX-2 in neutrophils. Immunofluorescence of COX-2 using human neutrophils (2 × 10^5^) stimulated Cr-LAAO (50 μg/mL), LPS (1 μg/mL; positive control) or RPMI (negative control) for 1 h at 37 °C and 5% CO_2_. This figure was created using images from Servier Medical Art Commons Attribution 3.0 Unported License (https://smart.servier.com) (**A**). Servier Medical Art by Servier is licensed under a Creative Commons Attribution 3.0 Unported License. The images were collected using constant automatic gain among the samples to quantify the differences in absolute levels of fluorescence intensity different conditions in 100 × magnification oil immersion objective. Figure representative of one experiment of three independent experiments (**B**). Analysis of the mean fluorescence intensity of COX-2 immunofluorescence was performed using 10 cells in field of view of each condition collected impartially (**C**) and plotted at the fluorescence intensity per cell (**D**). Values are mean S.E.M. from 3 donors. *P < 0.05, **P < 0.01, ***P < 0.001, ****P < 0.0001 compared to negative control (Data were presented with ANOVA followed by Dunnett post-test).

In order to show the presence of COX-2 in the cell cytoplasm, immunofluorescence experiments were conducted. Cells stimulated with Cr-LAAO or LPS had a higher fluorescence intensity after COX-2 labelling when compared to negative control (RPMI), confirming the results obtained with protein expression (Fig. 3).

### cPLA_2_-α activation are involved in increased lipid body formation

**Figure 4:**
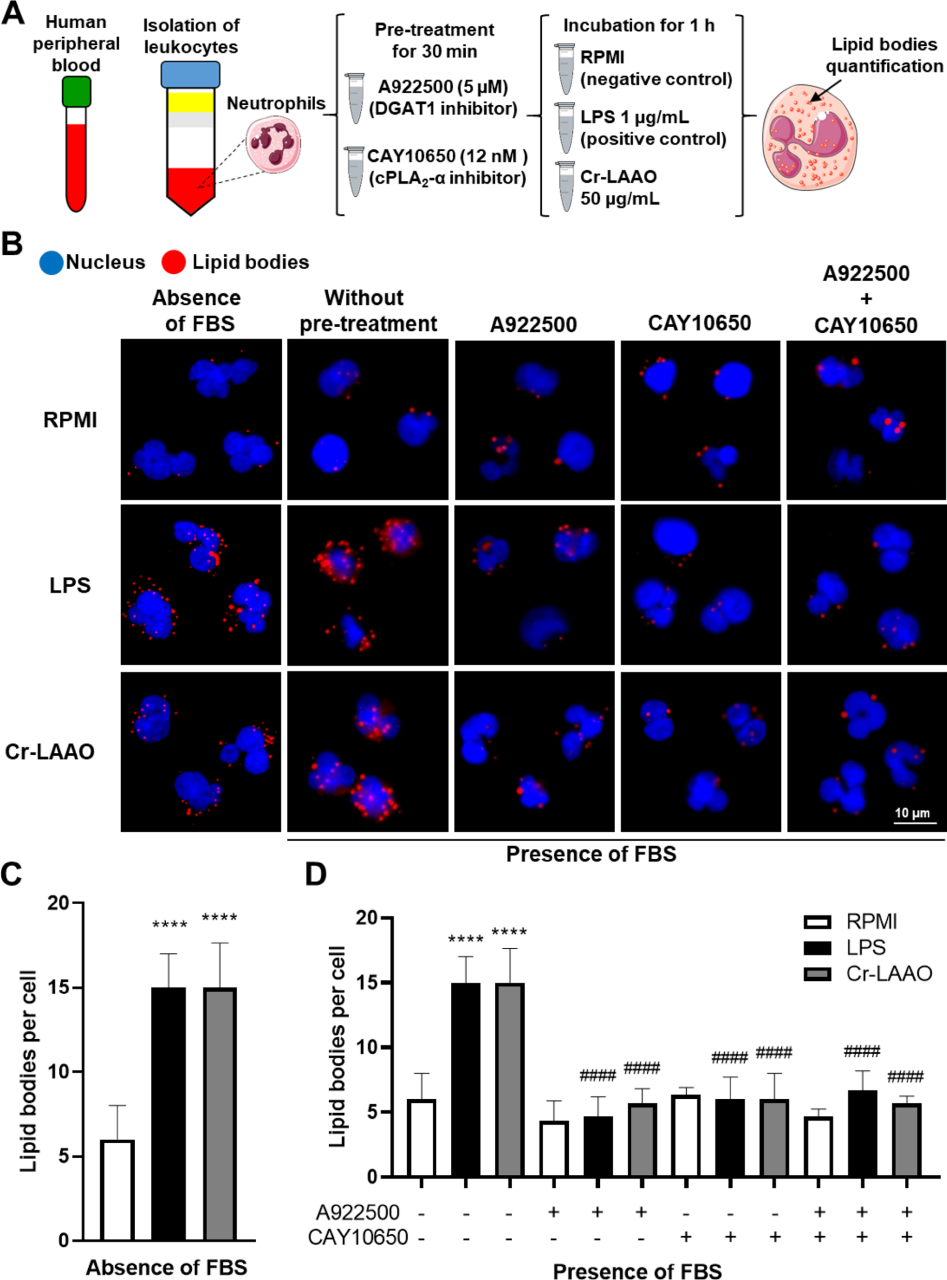
Production of lipid bodies in neutrophils pretreated with DGAT and cPLA_2_ inhibitor. LBs quantification stained with neutrophils (2 × 10^5^) pre-treated or not with inhibitors CAY10650 (cPLA_2_) and A922500 (DGAT) and stimulated Cr-LAAO (50 μg/mL), LPS (1 μg/mL; positive control) or RPMI (negative control) for 1 h at 37 °C and 5% CO_2_. This figure was created using images from Servier Medical Art Commons Attribution 3.0 Unported License (https://smart.servier.com) (**A**). Servier Medical Art by Servier is licensed under a Creative Commons Attribution 3.0 Unported License. Figure representative of one experiment of three independent experiments (**B**). The mean number of LBs from neutrophils stimulated in the absence of fetal bovine serum (FBS) (**C**) and in the presence of fetal bovine serum (**D**) was measured in 50 cells in the vision field of each condition impartially collected from 3 donors. Values are mean S.E.M. from 3 donors. *P < 0.05, **P < 0.01, ***P < 0.001, ****P < 0.0001 compared to negative control, and ^#^*P* < 0.05, ^##^*P* < 0.01, ^###^*P* < 0.001, ^####^*P* < 0.0001 compared to without pre-treatment with inhibitor (Data were presented with ANOVA followed by Tukey post-test).

cPLA_2_-α (CAY10650) and diacylglycerol acyltransferase 1 (DGAT1) (A922500) inhibitors were used to evaluate the mechanisms of Cr-LAAO-induced LB biogenesis. LB quantification demonstrated an increase in neutrophils stimulated for 2 h with Cr-LAAO or LPS (positive control) when compared to RPMI (negative control). However, when the specific inhibitors were used, together or separately, the LB levels were reduced to a baseline similar to the negative control group, demonstrating that the cPLA_2_ pathway was activated and participated in LB biogenesis in these cells in a mechanism that also involves DGAT-1 dependent lipid remodelling. In addition, the LBs formation in the culture medium without fetal bovine serum (FBS) was evaluated. It was observed that even in the absence of FBS there is an increase in LBs formation in human neutrophils stimulated with Cr-LAAO, while the negative control showed baseline levels of LBs formation in human neutrophils both in presence or absence of FBS (Fig. 4).

### Pharmacological treatment

**Figure 5:**
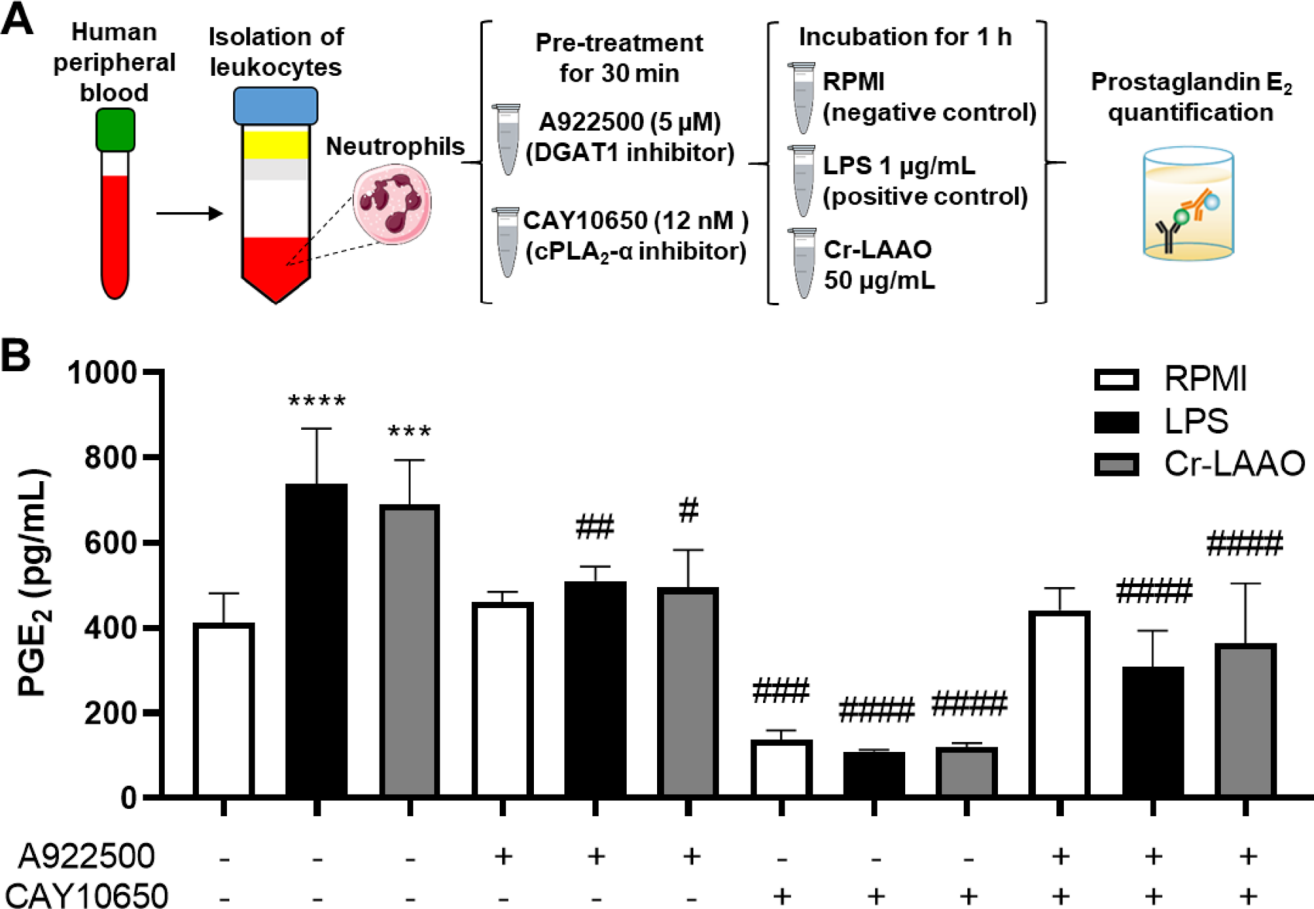
Prostaglandin E_2_ release by neutrophils pretreated with DGAT and cPLA2 inhibitor. PGE_2_ quantification in the supernatant of neutrophils (2 × 10^5^) pre-treated or not with CAY10650 (cPLA_2_) and A922500 (DGAT) inhibitors and followed by stimulation with Cr-LAAO (50 μg/mL), LPS (1 μg/mL; positive control) or RPMI (negative control) for 1 h at 37 °C and 5% CO_2_. This figure was created using images from Servier Medical Art Commons Attribution 3.0 Unported License (https://smart.servier.com) (**A**). Servier Medical Art by Servier is licensed under a Creative Commons Attribution 3.0 Unported License. PGE_2_ concentrations were quantitated by specific EIA in supernatant collected after incubation with RPMI or LPS or Cr-LAAO. The results were expressed as pg/mL of PGE_2_ produced and represent the mean ± S.E.M of 3 donors (**B**). *P < 0.05, **P < 0.01, ***P < 0.001, ****P < 0.0001 compared to negative control, and ^#^*P* < 0.05, ^##^*P* < 0.01, ^###^*P* < 0.001, ^####^*P* < 0.0001 compared to without pre-treatment with inhibitor (Data were presented with ANOVA followed by Tukey post-test).

cPLA_2_-α (CAY10650) and DGAT1 (A922500) inhibitors were used to characterize the mechanisms involved in the PGE_2_ release pathway. Cr-LAAO, similarly to LPS, stimulated PGE_2_ release compared to the negative control (RPMI) (Fig. 5). When neutrophils were treated with the DGAT inhibitor, we observed a reduction in PGE_2_ cell release after stimulation with Cr-LAAO and LPS, compared to cells without this treatment and with negative control cells. The treatment of neutrophils with a cPLA_2_-α inhibitor also showed reduced PGE_2_ in cells without treatment and in negative control cells. When both inhibitors were administered simultaneously, neutrophils incubated with Cr-LAAO or LPS showed reduced PGE_2_ release, demonstrating that Cr-LAAO and LPS used cPLA_2_ and lipid bodies to generate PGE_2_. We observed a greater effect of CAY10650 on inhibition of PGE_2_ release when used alone than when added in combination with the DAGT1 inhibitor (Fig. 5).

**Figure 6:**
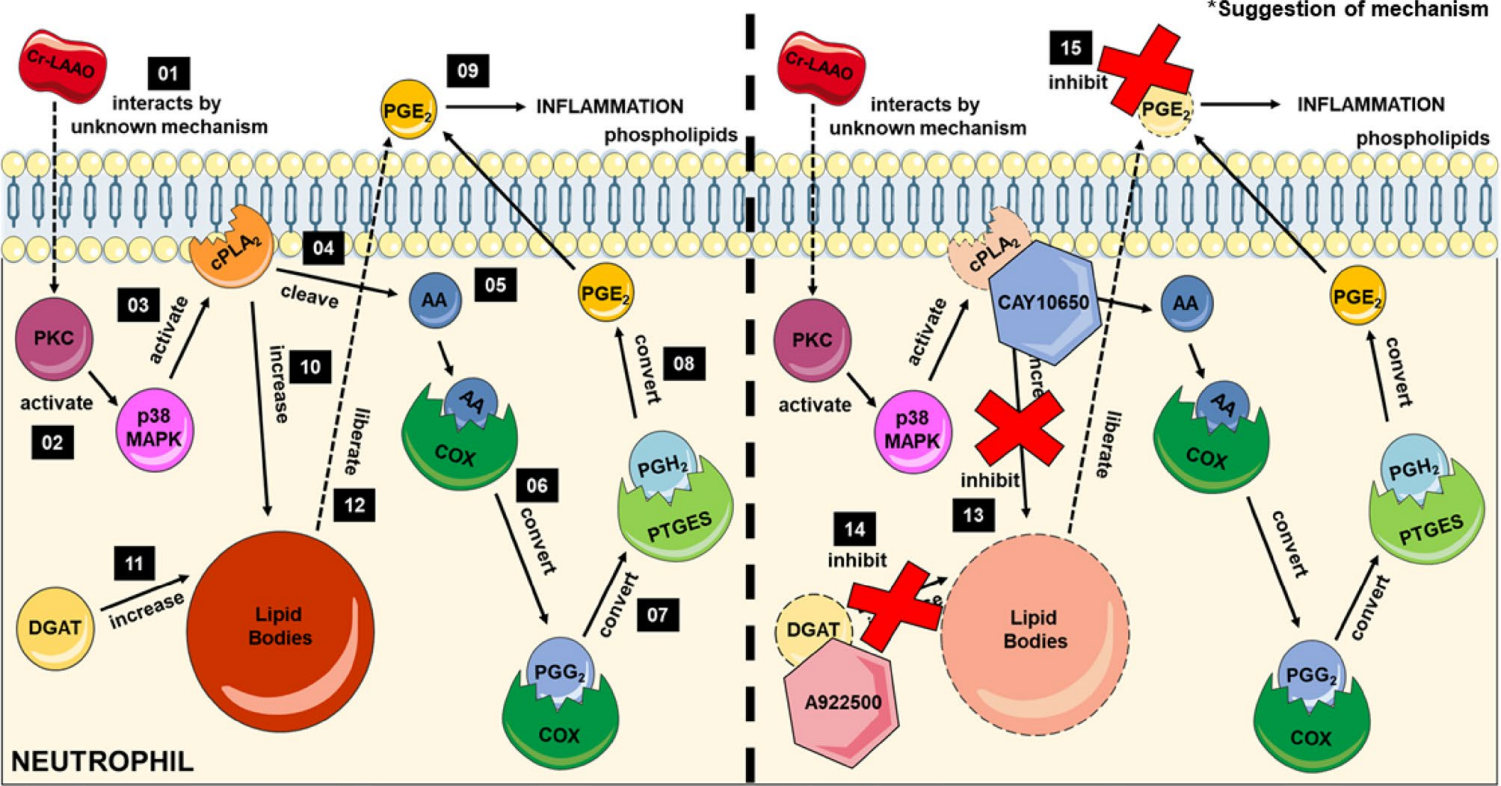
Suggestion of mechanism of activation of the cyclooxygenase pathway stimulated by Cr-LAAO. The representative scheme shows Cr-LAAO action on neutrophils. Firstly, Cr-LAAO interacts with the cellular membrane by an unknown mechanism (**01**), leading to PKC activation (**02**), which stimulates the p38 MAPK phosphorylation (**03**), and cPLA_2_ phosphorylation and activation (**04**). Activated cPLA_2_ cleaves membrane phospholipids to form arachidonic acid (AA) (**05**). AA can be catalyzed by COX-1 or COX-2 (**06**), forming PGH2, metabolized by PTGES (**07**) to form PGE_2_ (**08**) for release (**09**). Activation of cPLA_2_ (**10**) and DGAT (**11**) may lead to increased numbers of LBs in neutrophils because LB is rich in arachidonic acid (AA) for PGE_2_ synthesis. Cr-LAAO can utilize LBs content to release PGE_2_ (**12**). Thus, in the presence of cPLA_2_-α (CAY10650) (**13**) and DGAT (A922500) (**14**) inhibitors, there is a decrease in the PGE_2_ level released (**15**). This figure was created using images from Servier Medical Art Commons Attribution 3.0 Unported License (https://smart.servier.com). Servier Medical Art by Servier is licensed under a Creative Commons Attribution 3.0 Unported License.

When considered as a whole, the data obtained in this study and previous results published in the literature, we proposed a mechanism of action of Cr-LAAO on human neutrophils for PGE_2_ production. Firstly, Cr-LAAO interacts with the cellular membrane by an unknown mechanism, leading to PKC activation, which stimulates the p38 MAPK phosphorylation, and cPLA_2_ activation. Activated cPLA_2_ cleaves membrane phospholipids to form arachidonic acid (AA). AA can be catalyzed by COX-1 or COX-2, forming PGH_2_, metabolized by PTGES to form PGE_2_ for release. Activation of cPLA_2_ and DGAT may lead to increased numbers of lipid bodies in neutrophils. Cr-LAAO can utilize lipid bodies content to release PGE_2_. Thus, in the presence of cPLA_2_ (CAY10650) and DGAT (A922500) inhibitors, there is a decrease in the level of PGE_2_ released (Fig. 6).

## Discussion

In leukocytes, cell membranes are permeable to hydrogen peroxide, which can activate leukocyte functions. In neutrophils, hydrogen peroxide regulates both the extension of pseudopodia and the orientation and direction of the cell^32^. Moreover, there is evidence demonstrating that hydrogen peroxide controls the recruitment of leukocytes to the inflammatory site^33^. Klyubin et al.^34^ were the first to propose that hydrogen peroxide acts as a chemotactic in leukocytes^34^.

LAAO from snake venoms have been described to perform several biological functions, including proinflammatory activities^27^. According to previous studies, Cr-LAAO activates isolated human neutrophils and stimulates ROS production (hydrogen peroxide and superoxide anion), chemotaxis, phagocytosis, cytokine release such as IL-8, IL-6, TNF-α, and LTB_4_, as well as the release of PGE_2_, NETs, and MPO, in addition to activating p38MAPK and PI3K^28,29^. Moreover, Costa et al.^35^ recently reported the inflammatory responses induced by Cr-LAAO. The authors showed that this enzyme induced neutrophil recruitment and IL-6, IL-1β, LTB_4_, and PGE_2_ release by murine macrophages. Paloschi et al.^30^ showed that Cr-LAAO induces NADPH oxidase complex and PKC-α activation, which contributes to the ROS production (hydrogen peroxide and superoxide anion) observed earlier.

Studies from our laboratory showed that isolated human neutrophils stimulated with Cr-LAAO at several concentrations (6, 12.5, 25, 50, and 100 µg/mL) remained alive until 12 h of incubation, using MTT and trypan blue viability methods. Based on these results, we adopted a concentration equal to 50 μg/mL, an incubation period of 1 h, and a temperature of 37 °C in a humid atmosphere of 5% CO_2_^28,29^. Moreover, this period of time was defined based on PGE_2_ production by neutrophils under Cr-LAAO action, as previously reported by Pontes et al.^29^. However, the pathways responsible for this effect were not identified.

Microarray technology allows the investigation of thousands of genes simultaneously, substantially increasing the analytical capacity of molecular processes^36^. A study employing microarray assays with the LAAO of *Ophiophagus hannah* (OH-LAAO) on human breast adenocarcinoma cells (MCF-7) showed the expression of 178 genes after treatment with the enzyme, of which 27 were expressed due to the cytotoxic action of LAAO in relation to apoptosis, autophagy, cell cycle, DNA replication, oxidative stress, proteolysis, and intracellular signalling^37^. In another study, Guo et al.^38^ used microarray analysis to screen differentially expressed genes related to molecules involved in the TGF-β signalling pathway in human hepatocellular carcinoma (HepG2) cells in response to the action of LAAO from *Agkistrodon blomhoffii ussensis* (Akbu-LAAO)^38^. Pontes et al.^29^ have previously demonstrated PGE_2_ release by human neutrophils stimulated with Cr-LAAO; therefore, we decided to perform a microarray analysis on 38 genes related to COX signalling pathway and LBs, which may be involved in PGE_2_ production and release. These selected genes corroborate previous data obtained by Pontes et al.^29^ and complement the cellular activation mechanism of this enzyme on human neutrophils.

The COX pathway is initially activated by PLA_2_s, which perform various functions in the maintenance of homeostasis through membrane phospholipid cleavage to produce lipid mediators^39^. Cytosolic PLA_2_ (cPLA_2_) is a type of PLA_2_ that is activated upon phosphorylation (p-cPLA_2_)^39^. PLA2G4A is one of the genes encoding cPLA_2_-α, the cytosolic form prominently expressed in neutrophils^40^. cPLA_2_-α, translocates from the cytoplasm to the intracellular membrane in response to calcium to stimulate the release of arachidonic acid from these membranes. cPLA_2_-α is phosphorylated by MAPKs, which increases its catalytic activity^39,41^. Once activated, cPLA_2_-α releases arachidonic acid from the membrane phospholipids, which triggers the activity of enzymes that oxygenate arachidonic acid (AA), generating various eicosanoids^42^. During this process, AA is formed and subsequently metabolised by COXs, which depending on the stimulus, can be driven by COX-1 (constitutively active) or COX-2 (induced), resulting in PGE_2_ as the end product^9,10^. Studies have shown that the expression of the PLAA gene is necessary for the production of AA, leading to an increase in the production of PGE_2_ through the activation of cPLA_2_, iPLA, and COX-2, in response to stimuli such as TNF-α and LPS^43–45^.

In the present study, we observed an association between PLAA and cPLA_2_ gene expression and the COX-2 accumulation for PGE_2_ release. Stimulation of neutrophils with Cr-LAAO resulted in the expression of cPLA_2_ and accumulation of its phosphorylated form (p-cPLA_2_), demonstrating enzyme activation. Gene and protein expression for COX-1 showed that there was an expression of this constitutive protein in all groups. However, with respect to COX-2, there was a greater expression in neutrophils stimulated with Cr-LAAO and LPS when compared to the negative control, demonstrating COX-2 accumulation in the cytosol. COX-2 presence in the cytoplasm was confirmed with immunofluorescence imaging, demonstrated with an increase in the fluorescence level of the labeled enzyme in Cr-LAAO-stimulated neutrophil cytosol. In addition, the expression of PTGES, the protein responsible for the conversion of PGH_2_ to PGE_2_, was evaluated, showing the presence of this protein in neutrophils stimulated with Cr-LAAO. This is the first study to elucidate the mechanism underlying the cyclooxygenase pathway in human neutrophils stimulated with Cr-LAAO, which can be important for the comprehension and management of the local aspects observed in snakebites.

Diverse enzymes that lead to eicosanoid biosynthesis may be associated with LBs under activation conditions, including cPLA_2_-α^46,47^. cPLA_2_-α can remodel phospholipids of the endoplasmic reticulum and increase LBs involving deacylation/reacylation reactions^48–50^. Gubern et al.^51^ demonstrated that cPLA_2_-α inhibition reduces the levels of LBs in CHO-K1 cells. The authors also showed that knocking down cPLA_2_-α expression with short interfering RNA is similar to pharmacological inhibition in terms of enzyme activity and LBs biogenesis^51^. The protein expression results showed that there was no expression of p-cPLA2-α in the cells pretreated with CAY10650. Similarly, this result was observed when AACOCF3, another effective cPLA2-α inhibitor^52–55^, was used. Meanwhile I take this opportunity to bring to the knowledge of that AACOCF3 stimulated the LBs formation in neutrophils as demonstrated by Bozza et al.^56^.

LB biogenesis during inflammation is regulated both by the stimulus that the cell is submitted to and by specific signalling pathways. Naïve leukocytes, including neutrophils, have few LBs in their cytoplasm. However, when cells are activated, there is a significant increase in the number and size of LBs^31^. Nose et al.^57^ demonstrated that perilipin 3 (PLIN3) plays a crucial role in the formation of LBs in neutrophils for the release of PGE_2_. In our study, we found a positive regulation of PLIN3 and PLIN2 in human neutrophils after stimulation with Cr-LAAO. Among the proteins that comprise the LBs, PLIN2 is one of the main structural proteins found in all cell types^58^, which was a relevant factor in this study to examine PLIN2. Chen et al.^59^ showed that the increase in PLIN2 mRNA level is directly linked to increased mRNA levels of proinflammatory cytokines, such as TNF-α and IL-6. This finding corroborates the results obtained by Pontes et al.^28,29^ which showed that Cr-LAAO induces TNF-α and IL-6 release by neutrophils. Gene expression results obtained in the current study showed that there was an increase in PLIN2 expression in neutrophils stimulated with Cr-LAAO, supporting previous findings.

Lipid bodies are intracellular organelles that mainly store triacylglycerols (TGs) and sterol esters (SEs) as a bioenergy source. Harris et al.^60^ showed that DGAT1 and DGAT2 are responsible for the synthesis of almost all TGs in adipocytes, because in the absence of DGAT, adipocytes lack TGs and LBs, but in macrophages, they are not absolutely necessary for the formation of LBs. DGAT1 is proposed to possess dual topology, contributing to triacylglyceride (TAG) synthesis on both sides of the endoplasmic reticulum membrane and esterifying only the pre-formed fatty acids. Studies suggest that DGAT2 translocates to LBs and is associated with other structural proteins to synthesis TGs from endogenous and exogenous fatty acids^56,61^. In this study, microarray data demonstrated for the first time the signalling dependent up-regulation of DGAT1 and DGAT2 in human neutrophils. In addition, an increase in the amount of LBs present in the neutrophil cytoplasm stimulated with Cr-LAAO was verified. Treatment with a DGAT1 inhibitor (A922500) diminished the biogenesis of LBs to baseline numbers in neutrophils stimulated with Cr-LAAO. This data is in agreement with previous results that demonstrate that TAG is the main component in LB and consistent remodeling of AA pools from human neutrophil^57^.

Previous studies have shown that AA-rich LBs rapidly associate with phagosomes, suggesting that AA derived from LBs functions as an activator of the NADPH oxidase complex in phagosomes^62,63^. According to Paloschi et al.^30^, NADPH oxidase is activated by Cr-LAAO in human neutrophils. Additionally, as reported by Pontes et al.^29^, these cells phagocytose more zymosan particles under Cr-LAAO stimulation. Therefore, the present data support that LB biogenesis could be mediated by the activation of DGAT and cPLA_2_ in neutrophils stimulated with Cr-LAAO, and it is possible that the increase in LBs may participate in the activation of NADPH oxidase, as shown previously by Paloschi et al.^30^.

Other studies conducted with snake venoms and isolated toxins have demonstrated the production of LBs in macrophages. De Carvalho et al.^64^ investigated the venom capacity of *Crotalus durissus ruruima* and an isolated phospholipase A_2_ (CBr) to activate macrophages. The researchers focused on lipid droplet formation and the synthesis of lipid mediators involved in these effects, demonstrating PLIN2 recruitment, as well as the expression and presence of PGE_2_ in LBs as sites for prostatenoid synthesis. The isolated phospholipase A_2_ from *Crotalus durissus terrificus* venom, which has an anti-inflammatory effect, caused increased LB and PLIN2 recruitment in macrophages^65^. The increase in LB formation in macrophages from J774 cell line has also been observed upon stimulation with two basic myotoxic phospholipase A_2_: BaTX-I, a catalytically inactive Lys-49 variant, and BaTX-II, a catalytically active Asp-49, and of one acidic myotoxic PLA_2_, the BaPLA_2_, a catalytically active Asp-49, all of them isolated from *Bothrops atrox* snake venom^66^. MT-II, a Lys49-PLA_2_ from *Bothrops asper* venom devoid of catalytic activity, induces the formation of LBs and the synthesis of PGE_2_, as well as its localisation in LBs^67^. In addition, a study on the function of MT-III, a sPLA_2_ from *B. asper*, in the biogenesis of LBs in murine macrophages, demonstrated the expression of PLIN2, which depends on the PKC, PI3K, p38MAPK, ERK1/2, cPLA_2_, and iPLA_2_ signalling pathways, but not in the PTK, COX-1, or COX-2 pathways^68^. In human neutrophils, the relation between cPLA_2_ and LB biogenesis is unknown. In the present study we showed that Cr-LAAO could activate cPLA_2_ in neutrophils to induce an increase in the LBs formation in the cell. This accumulation of LBs may be directly related to the release of PGE_2_, a product of COX-2 activation, which has been shown to accumulate in the cytosol of activated neutrophils, together with PLIN2 recruitment. In addition to this finding, we have seen in our previous studies the presence of important signalling proteins for this process, such as PKC, PI3K and p38MAPK^28–30^.

Collectively, the data of the present study demonstrate for first time that Cr-LAAO induces the regulation of lipid signalling and metabolism in human neutrophils, leading to increased LB biogenesis and PGE_2_ production. Moreover, Paloschi et al.^30^ and Pontes et al.^29^ demonstrated that Cr-LAAO induces the activation of PKC and p38-MAPK, respectively; and here, we demonstrated that Cr-LAAO induces the cPLA_2_ activation which present an important role in the mechanisms of LBs biogenesis in human neutrophils (Fig. 6). The inhibition of LB formation by DGAT-1 inhibitor also lead to decrease the PGE_2_ production suggesting that LB participate in the increase of the PGE_2_ synthesis in Cr-LAAO-stimulated neutrophils. However, the data shown here do not rule out the possibility that LB formation and PGE_2_ release occur simultaneously and independently of each other. Although the mechanism through which Cr-LAAO interacts with the cellular membrane for neutrophil activation is still not fully understood, Cr-LAAO regulates lipid metabolism and signalling in human neutrophils through the expression and activation of enzymes and structural proteins, including cPLA_2_, DGAT-1, COX-2, PGES and PLIN2 and PLIN3, that participates in the amplification of the inflammatory process by triggering intracellular signalling cascade that culminate in lipid body formation and increased PGE_2_ synthesis.

## Methods

### Chemicals and reagents

Crystallized *Calloselasma rhodostoma* venom, lipopolysaccharides from *Escherichia coli* O111:B4 (LPS), Histopaque 1077, 3,30-diaminobenzidine tablets, hydrogen peroxide 30%, ethylene glycol-bis (β-aminoethyl ether)-*N*,*N*,*N*′,*N*′-tetraacetic acid (EGTA), HEPES, Triton X-100, leupeptin, aprotinin, phenylmethylsulfonyl fluoride (PMSF), sodium orthovanadate, protease and phosphatase inhibitor cocktail, ethylenediaminetetraacetic acid disodium salt dihydrate (Na_2_ EDTA), bicinchoninic acid protein assay Kit (BCA), oil Red O, AACOCF3 (arachidonyl trifluoromethyl ketone), anti-mouse Fab specific-FITC and anti-β-actin were purchased from Sigma Chem. Co. (Misssouri, USA). CAY10650, A922500, prostaglandin E_2_ ELISA Kit, anti-COX-1 and anti-COX-2 were purchased from Cayman Chemical (Michigan, USA). Anti-cPLA_2_-α, anti-PTGES and anti-p-cPLA_2_-α were purchased from Santa Cruz Biotechnology (Texas, USA). PureLink kit, SuperScript III Reverse Transcriptase, GeneChip WT PLUS Reagent Kit and GeneChip Clariom S Array Human were purchased from Thermo Fisher Scientific (Massachusetts, USA). iTaq Universal SYBR Green Supermix were purchased from Bio-Rad (California, USA). All salts and reagents used obtained from Merck Millipore (Darmstadt, Germany) with low endotoxin or endotoxin-free grades.

### Isolation and biochemical characterization of Cr-LAAO

Cr-LAAO was isolated according to Pontes et al.^28^. In brief, *Calloselasma rhodostoma* venom (30 mg) was dissolved in 1.0 mL of 0.02 M Tris–HCl buffer, pH 8.0, centrifuged at 755* g* for 10 min at room temperature and the clear supernatant applied on a 70 cm × 0.9 cm Superdex G-75 column, which was previously equilibrated and then eluted with the same buffer. The fraction I showing LAAO activity was lyophilized, diluted with 0.02 M Tris–HCl buffer, pH 8.0 and then applied on a 4.0 × 0.6 cm Q-Sepharose Fast Flow column (GE Healthcare), previously equilibrated with the same buffer, using a crescent concentration NaCl gradient (0–100%). The activity of l-amino acid oxidase was performed according to Paloschi et al.^30^ using 50 µg/mL of Cr-LAAO added to the mixture containing horseradish peroxidase (50 µg/mL), 100 mM l-leucine, 10 mM 3′3′-diaminobenzidine in 100 mM Tris–HCl buffer (pH 7.8), incubated at 37 °C for 30 min. The reaction was stopped using a solution of 10% citric acid and the absorbance was measured on a spectrophotometer (Synergy HT, Biotek) at 490 nm.

### Neutrophil isolation

Peripheral blood neutrophils were obtained from self reportedly healthy donators (18–40 years old). Informed consents were obtained at the time of the blood draw. All experiments were performed in accordance with relevant guidelines and regulations. All participants gave informed consent prior to their inclusion in the study, and the Brazilian IRB (Institutional Review Board) of the Center of Tropical Medicine Research (CEPEM, Rondônia, Brazil—Approval Number 1.739.023) approved it. In brief, blood was collected in vacuum tubes containing heparin and diluted in phosphate buffered saline (PBS, 14 mM NaCl, 2 mM NaH_2_PO_4_·H_2_O, 7 mM Na_2_HPO_4_·12H_2_O), pH 7.4, after local asepsis. For the separation of leukocytes, Histopaque 1077 was added to the tubes and then the diluted blood was carefully added over the reagent. After centrifugation at 400×*g* for 30 min, neutrophils were collected from the bottom of the tube, along with the erythrocytes and were transferred to another tube, according to Pontes et al.^28,29^. Lysis of red blood cells was performed using lysis buffer (0.15 M NH_4_Cl, 0.01 M KHCO_3_, 0.0001 M Na_2_ EDTA), homogenized and subjected to a temperature of − 20 °C for 5 min, and then centrifuged. Neutrophils were washed with PBS and an aliquot of isolated neutrophils was used for determining the total number of neutrophils in a Neubauer’s chamber after cell staining (1:20, v/v) with Turk solution (violet crystal 0.2% in acetic acid 30%). The purity of the isolated cell population was determined by Panotic staining of cytospin preparations and by flow cytometry analysis (FACScan). The average purity achieved by our isolation technique was 99% of the neutrophils^28^.

### Neutrophil activation

Neutrophils isolated according to item above from 3 different donors, were suspended in a RPMI assay medium [RPMI-1640 medium supplemented with 100 mg/mL of gentamicin, 2 mM of l-glutamine and 2% of fetal bovine serum (FBS)]. Then, they were plated and incubated with RPMI (negative control), LPS (1 µg/mL; positive control) or Cr-LAAO (50 µg/mL) at 37 °C, in a humidified atmosphere (5% CO_2_) for 1 h.

### Gene expression

Human neutrophil mRNA (5 × 10^6^) isolated and stimulated for 1 h was extracted for microarray and RT-qPCR (Reverse transcription-quantitative polymerase chain reaction) using the PureLink kit (Thermo Fisher Scientific) according to manufacturer’s instructions. The cDNA for RT-qPCR was generated from 1 μg of total RNA using the SuperScript III Reverse Transcriptase kit (Thermo Fisher Scientific) and random primers according to the manufacturer’s instructions. Part of the extracted RNA was treated for microarray expression using the GeneChip WT PLUS Reagent Kit (Applied Biosystems, Thermo Fisher Scientific) for purification, reverse transcription, fragmentation and labeling of the samples, for application in a GeneChip Clariom S Array Human (Applied Biosystems, Thermo Fisher Scientific) according to the manufacturer's instructions. The microarray genes selected for this study are in Table S1. RT-qPCR was performed using the iTaq Universal SYBR Green Supermix kit (Bio-Rad) on Rotor-Gene Q (QIAGEN), with primers pre-designed for mRNA gene expression (DNA Express Biotechnology) analysis (Table S2). The relative fold change quantification of each gene was calculated by the ^2^ΔΔCt method^69^ using the reference gene hemoglobin subunit beta (HBB) for normalization.

### Western blot

For this assay, 1 × 10^7^ isolated and stimulated human neutrophils for 1 h according to items above pre-treated with CAY10650 (cPLA_2_-α inhibitor, 12 nM for 30 min^70^) or AACOCF3 (cPLA_2_ inhibitor, 20 µM for 30 min^71^) or the same vehicle used to dissolve the inhibitors in RPMI (control). For β-actin, cPLA_2_-α, p-cPLA_2_-α, COX-1, COX-2 and PTGES determinations, total protein extracts were prepared, resolved by 10% SDS/PAGE and transferred onto a PVDF membrane (Hybond, Amersham Pharmacia Biotech). Immunoblotting was performed using monoclonal antibodies to the referent proteins (Fig. S1). Blots were developed with 3,30-diaminobenzidine tablets and hydrogen peroxide (Sigma-Fast)^29^. The relative immunoreactivity bands of three independent experiments were quantified by densitometry using Image Studio Lite Ver 5.2 (LI-COR, Lincoln, Nebraska, EUA). The mean densitometry values of tested proteins were divided by the mean densitometry values of respective β-actin values to show the relative expression of each protein as a ration mean of the protein/β-actin^30^.

### Immunofluorescence

For immunofluorescence microscopy, 2 × 10^5^ isolated and stimulated human neutrophils as mentioned above for 1 h were seeded on 70% alcohol-washed coverslips and treated with Poly-l-Lysine (Sigma Aldrich) and placed in 24-well plates. The cells were fixed with 4% paraformaldehyde at room temperature for 15 min. Next, cells were permeabilized with acetone PA for 5 min at room temperature and the cells were incubated with the anti-COX-2 primary antibody (Cayman Chemical) overnight, followed by incubation with FITC-conjugated Fab anti-mouse secondary antibody (Sigma Aldrich) 1 h and staining with Alexa Fluor 647 phalloidin (Invitrogen), according to the manufacturer's instructions. After DAPI staining, the coverslips were mounted with Fluoromout G (Sigma Aldrich) and analyzed under a Nikon Eclipse 80i microscope with a 100 × magnification oil immersion objective. The images were collected using constant automatic gain among the samples to quantify the differences in absolute levels of fluorescence intensity different conditions. Ten fields of view of each condition were collected impartially. The acquired images were subsequently analyzed using ImageJ software (National Institutes of Health) to quantify the absolute total fluorescence intensity. The calculated fluorescence intensity of the fields of view was plotted as mean normalized intensity for the total number of cells^72^.

### Lipid bodies quantification

The 0.3% Oil Red O (ORO) solution was prepared in isopropanol P.A., diluted in distilled water (3:2) and filtered through a paper filter to avoid precipitates. Isolated and stimulated neutrophils (2 × 10^5^) for 2 h in the presence or absence of inhibitors CAY10650 (cPLA_2_-α inhibitor, 12 nM for 30 min^70^) and A922500 (DGAT1 inhibitor; 5 µM for 30 min^73^), were seeded on 70% alcohol-washed coverslips, treated with Poly-l-Lysine (Sigma Aldrich) and placed in 24-well plates. The supernatant was removed and the cells were fixed with 4% paraformaldehyde at room temperature for 15 min. The coverslips were stained with ORO (300 μL/well) for 2 min, washed with 30% isopropanol and subsequently with distilled water. The coverslips were mounted with Fluoroshild with DAPI (Sigma Aldrich) and analyzed under a Nikon Eclipse 80i microscope with a 100 × magnification oil immersion objective. LBs were quantified from fifty consecutive cells and the results were expressed of mean normalized for the total number of cells^74^.

### Prostaglandin E_2_ (PGE_2_) assay

PGE_2_ concentrations were measured in the supernatant of neutrophils (2 × 10^5^ cells) suspended in assay RPMI. Briefly, isolated and stimulated neutrophils were incubated with assay medium (negative control), LPS (1 μg/mL; positive control) or Cr-LAAO (50 μg/mL) diluted in assay medium for 1 h at 37 °C in a humidified atmosphere (5%CO_2_) in the presence or absence of inhibitors CAY10650 (cPLA_2_-α inhibitor, 12 nM for 30 min^66^) and A922500 (DGAT1 inhibitor; 5 µM for 30 min^73^). PGE_2_ concentrations in the supernatant were determined by a specific enzymatic immunoassay (EIA) previously described by Pontes et al.^29^ using a commercial kit (Cayman Chemicals).

### Statistical analysis

The graphs were plotted using GraphPad Prism Ver 7.04 (GraphPad Software Incorporated). The means and standard error of the mean (S.E.M.) of all data were obtained and compared by one-way ANOVA followed by Dunnett or Tukey post-test with significance probability levels less than 0.05. Individual comparisons using t-test was also checked.

## Supplementary information

Supplementary file
