# Supplementary material for: Cytosolic phospholipase A2-α participates in lipid body formation and PGE2 release in human neutrophils stimulated with an l-amino acid oxidase from Calloselasma rhodostoma venom
Source: Sci Rep. 2020 Jul 3;10:10976. doi: 10.1038/s41598-020-67345-3 (PMC7334223; doi:10.1038/s41598-020-67345-3)
Supplement: Supplementary file 1 — Supplementary file [file 41598_2020_67345_MOESM1_ESM.pptx]

## Slide 1
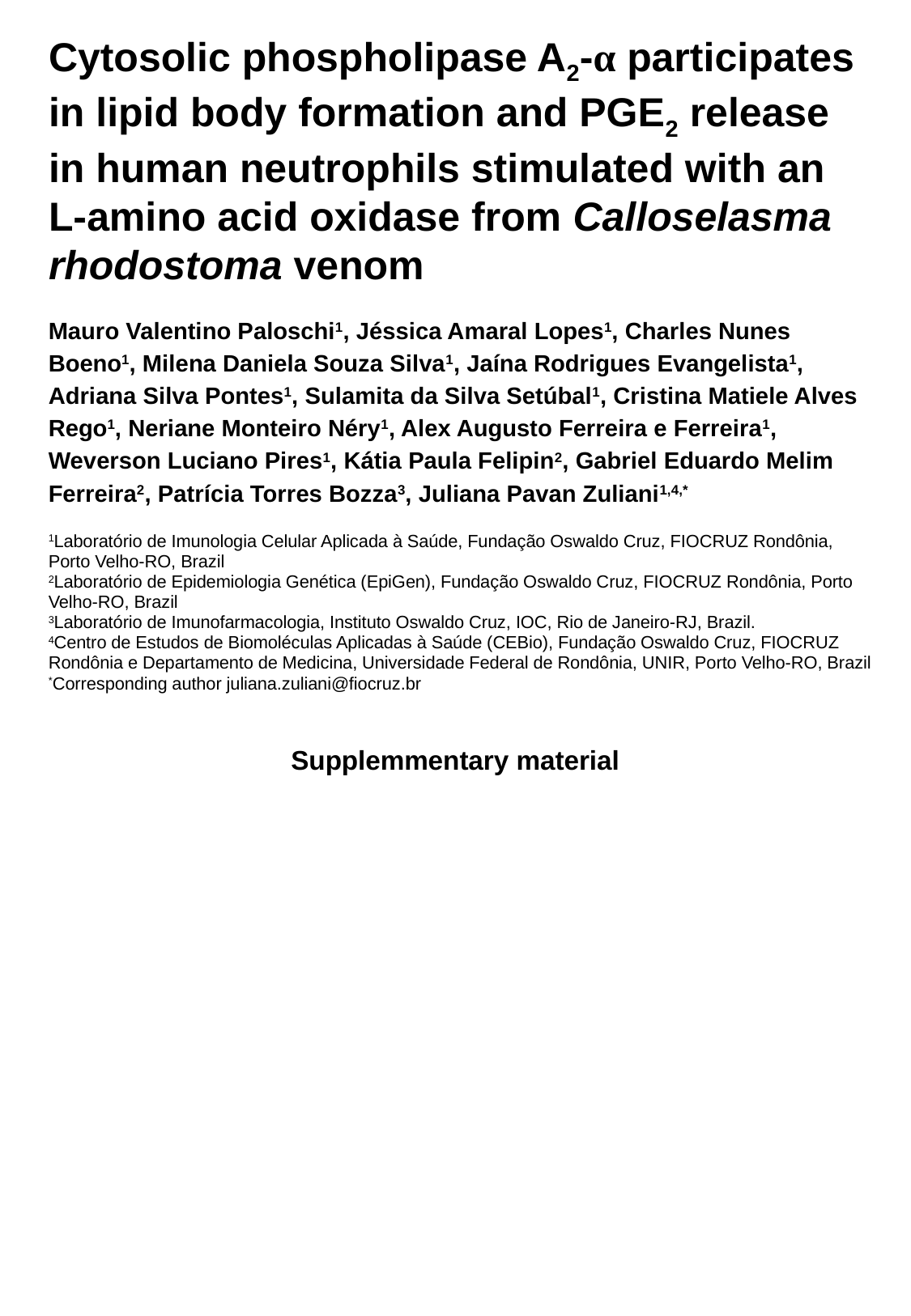

Cytosolic phospholipase A2-α participates in lipid body formation and PGE2 release in human neutrophils stimulated with an L-amino acid oxidase from Calloselasma rhodostoma venom
Mauro Valentino Paloschi1, Jéssica Amaral Lopes1, Charles Nunes Boeno1, Milena Daniela Souza Silva1, Jaína Rodrigues Evangelista1, Adriana Silva Pontes1, Sulamita da Silva Setúbal1, Cristina Matiele Alves Rego1, Neriane Monteiro Néry1, Alex Augusto Ferreira e Ferreira1, Weverson Luciano Pires1, Kátia Paula Felipin2, Gabriel Eduardo Melim Ferreira2, Patrícia Torres Bozza3, Juliana Pavan Zuliani1,4,*
1Laboratório de Imunologia Celular Aplicada à Saúde, Fundação Oswaldo Cruz, FIOCRUZ Rondônia, Porto Velho-RO, Brazil
2Laboratório de Epidemiologia Genética (EpiGen), Fundação Oswaldo Cruz, FIOCRUZ Rondônia, Porto Velho-RO, Brazil
3Laboratório de Imunofarmacologia, Instituto Oswaldo Cruz, IOC, Rio de Janeiro-RJ, Brazil.
4Centro de Estudos de Biomoléculas Aplicadas à Saúde (CEBio), Fundação Oswaldo Cruz, FIOCRUZ Rondônia e Departamento de Medicina, Universidade Federal de Rondônia, UNIR, Porto Velho-RO, Brazil
*Corresponding author juliana.zuliani@fiocruz.br
Supplemmentary material

## Slide 2
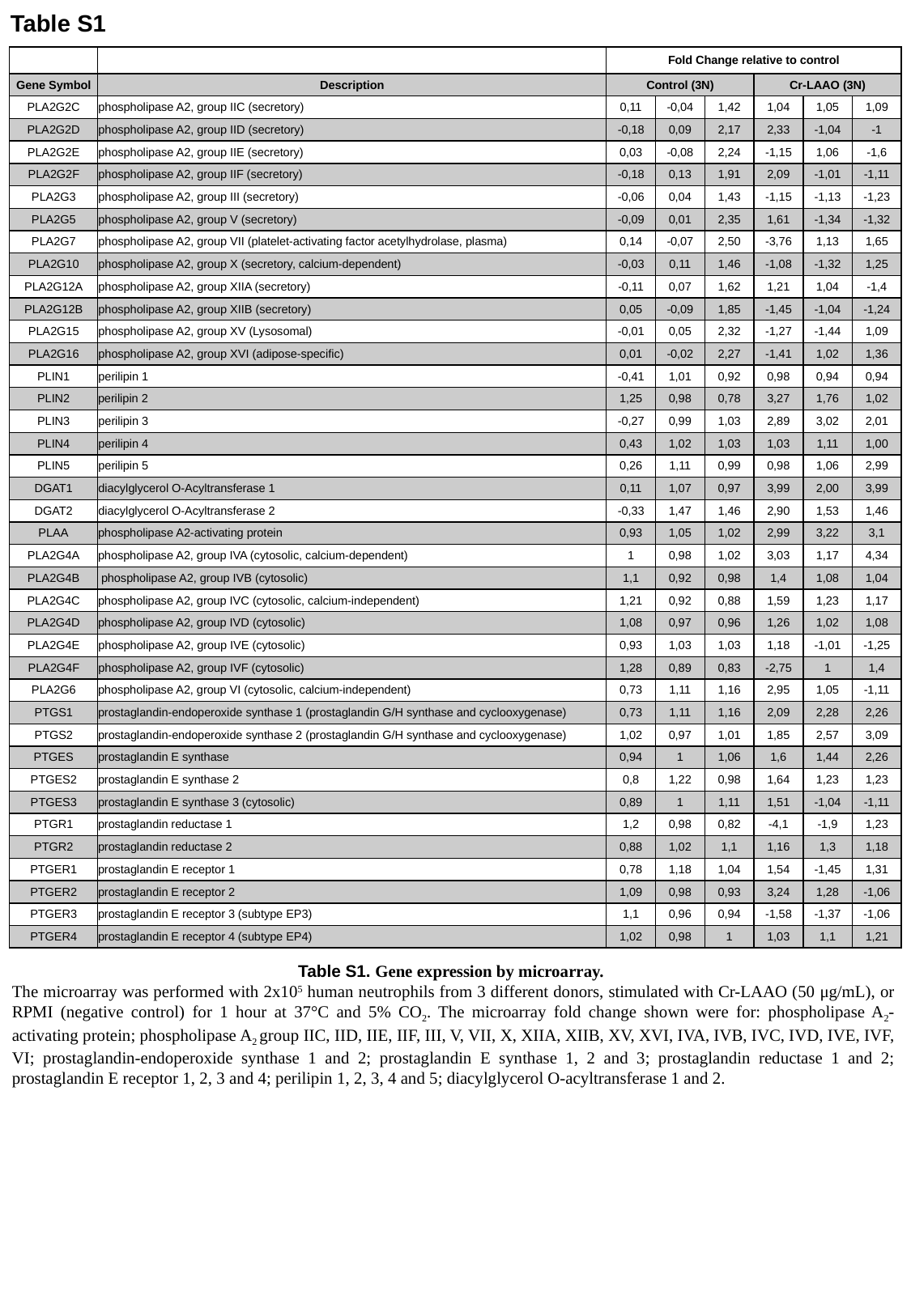

Table S1
| | | Fold Change relative to control | | | | | |
| --- | --- | --- | --- | --- | --- | --- | --- |
| Gene Symbol | Description | Control (3N) | | | Cr-LAAO (3N) | | |
| PLA2G2C | phospholipase A2, group IIC (secretory) | 0,11 | -0,04 | 1,42 | 1,04 | 1,05 | 1,09 |
| PLA2G2D | phospholipase A2, group IID (secretory) | -0,18 | 0,09 | 2,17 | 2,33 | -1,04 | -1 |
| PLA2G2E | phospholipase A2, group IIE (secretory) | 0,03 | -0,08 | 2,24 | -1,15 | 1,06 | -1,6 |
| PLA2G2F | phospholipase A2, group IIF (secretory) | -0,18 | 0,13 | 1,91 | 2,09 | -1,01 | -1,11 |
| PLA2G3 | phospholipase A2, group III (secretory) | -0,06 | 0,04 | 1,43 | -1,15 | -1,13 | -1,23 |
| PLA2G5 | phospholipase A2, group V (secretory) | -0,09 | 0,01 | 2,35 | 1,61 | -1,34 | -1,32 |
| PLA2G7 | phospholipase A2, group VII (platelet-activating factor acetylhydrolase, plasma) | 0,14 | -0,07 | 2,50 | -3,76 | 1,13 | 1,65 |
| PLA2G10 | phospholipase A2, group X (secretory, calcium-dependent) | -0,03 | 0,11 | 1,46 | -1,08 | -1,32 | 1,25 |
| PLA2G12A | phospholipase A2, group XIIA (secretory) | -0,11 | 0,07 | 1,62 | 1,21 | 1,04 | -1,4 |
| PLA2G12B | phospholipase A2, group XIIB (secretory) | 0,05 | -0,09 | 1,85 | -1,45 | -1,04 | -1,24 |
| PLA2G15 | phospholipase A2, group XV (Lysosomal) | -0,01 | 0,05 | 2,32 | -1,27 | -1,44 | 1,09 |
| PLA2G16 | phospholipase A2, group XVI (adipose-specific) | 0,01 | -0,02 | 2,27 | -1,41 | 1,02 | 1,36 |
| PLIN1 | perilipin 1 | -0,41 | 1,01 | 0,92 | 0,98 | 0,94 | 0,94 |
| PLIN2 | perilipin 2 | 1,25 | 0,98 | 0,78 | 3,27 | 1,76 | 1,02 |
| PLIN3 | perilipin 3 | -0,27 | 0,99 | 1,03 | 2,89 | 3,02 | 2,01 |
| PLIN4 | perilipin 4 | 0,43 | 1,02 | 1,03 | 1,03 | 1,11 | 1,00 |
| PLIN5 | perilipin 5 | 0,26 | 1,11 | 0,99 | 0,98 | 1,06 | 2,99 |
| DGAT1 | diacylglycerol O-Acyltransferase 1 | 0,11 | 1,07 | 0,97 | 3,99 | 2,00 | 3,99 |
| DGAT2 | diacylglycerol O-Acyltransferase 2 | -0,33 | 1,47 | 1,46 | 2,90 | 1,53 | 1,46 |
| PLAA | phospholipase A2-activating protein | 0,93 | 1,05 | 1,02 | 2,99 | 3,22 | 3,1 |
| PLA2G4A | phospholipase A2, group IVA (cytosolic, calcium-dependent) | 1 | 0,98 | 1,02 | 3,03 | 1,17 | 4,34 |
| PLA2G4B | phospholipase A2, group IVB (cytosolic) | 1,1 | 0,92 | 0,98 | 1,4 | 1,08 | 1,04 |
| PLA2G4C | phospholipase A2, group IVC (cytosolic, calcium-independent) | 1,21 | 0,92 | 0,88 | 1,59 | 1,23 | 1,17 |
| PLA2G4D | phospholipase A2, group IVD (cytosolic) | 1,08 | 0,97 | 0,96 | 1,26 | 1,02 | 1,08 |
| PLA2G4E | phospholipase A2, group IVE (cytosolic) | 0,93 | 1,03 | 1,03 | 1,18 | -1,01 | -1,25 |
| PLA2G4F | phospholipase A2, group IVF (cytosolic) | 1,28 | 0,89 | 0,83 | -2,75 | 1 | 1,4 |
| PLA2G6 | phospholipase A2, group VI (cytosolic, calcium-independent) | 0,73 | 1,11 | 1,16 | 2,95 | 1,05 | -1,11 |
| PTGS1 | prostaglandin-endoperoxide synthase 1 (prostaglandin G/H synthase and cyclooxygenase) | 0,73 | 1,11 | 1,16 | 2,09 | 2,28 | 2,26 |
| PTGS2 | prostaglandin-endoperoxide synthase 2 (prostaglandin G/H synthase and cyclooxygenase) | 1,02 | 0,97 | 1,01 | 1,85 | 2,57 | 3,09 |
| PTGES | prostaglandin E synthase | 0,94 | 1 | 1,06 | 1,6 | 1,44 | 2,26 |
| PTGES2 | prostaglandin E synthase 2 | 0,8 | 1,22 | 0,98 | 1,64 | 1,23 | 1,23 |
| PTGES3 | prostaglandin E synthase 3 (cytosolic) | 0,89 | 1 | 1,11 | 1,51 | -1,04 | -1,11 |
| PTGR1 | prostaglandin reductase 1 | 1,2 | 0,98 | 0,82 | -4,1 | -1,9 | 1,23 |
| PTGR2 | prostaglandin reductase 2 | 0,88 | 1,02 | 1,1 | 1,16 | 1,3 | 1,18 |
| PTGER1 | prostaglandin E receptor 1 | 0,78 | 1,18 | 1,04 | 1,54 | -1,45 | 1,31 |
| PTGER2 | prostaglandin E receptor 2 | 1,09 | 0,98 | 0,93 | 3,24 | 1,28 | -1,06 |
| PTGER3 | prostaglandin E receptor 3 (subtype EP3) | 1,1 | 0,96 | 0,94 | -1,58 | -1,37 | -1,06 |
| PTGER4 | prostaglandin E receptor 4 (subtype EP4) | 1,02 | 0,98 | 1 | 1,03 | 1,1 | 1,21 |
Table S1. Gene expression by microarray.
The microarray was performed with 2x10^5^ human neutrophils from 3 different donors, stimulated with Cr-LAAO (50 μg/mL), or RPMI (negative control) for 1 hour at 37°C and 5% CO2. The microarray fold change shown were for: phospholipase A2-activating protein; phospholipase A2 group IIC, IID, IIE, IIF, III, V, VII, X, XIIA, XIIB, XV, XVI, IVA, IVB, IVC, IVD, IVE, IVF, VI; prostaglandin-endoperoxide synthase 1 and 2; prostaglandin E synthase 1, 2 and 3; prostaglandin reductase 1 and 2; prostaglandin E receptor 1, 2, 3 and 4; perilipin 1, 2, 3, 4 and 5; diacylglycerol O-acyltransferase 1 and 2.

## Slide 3
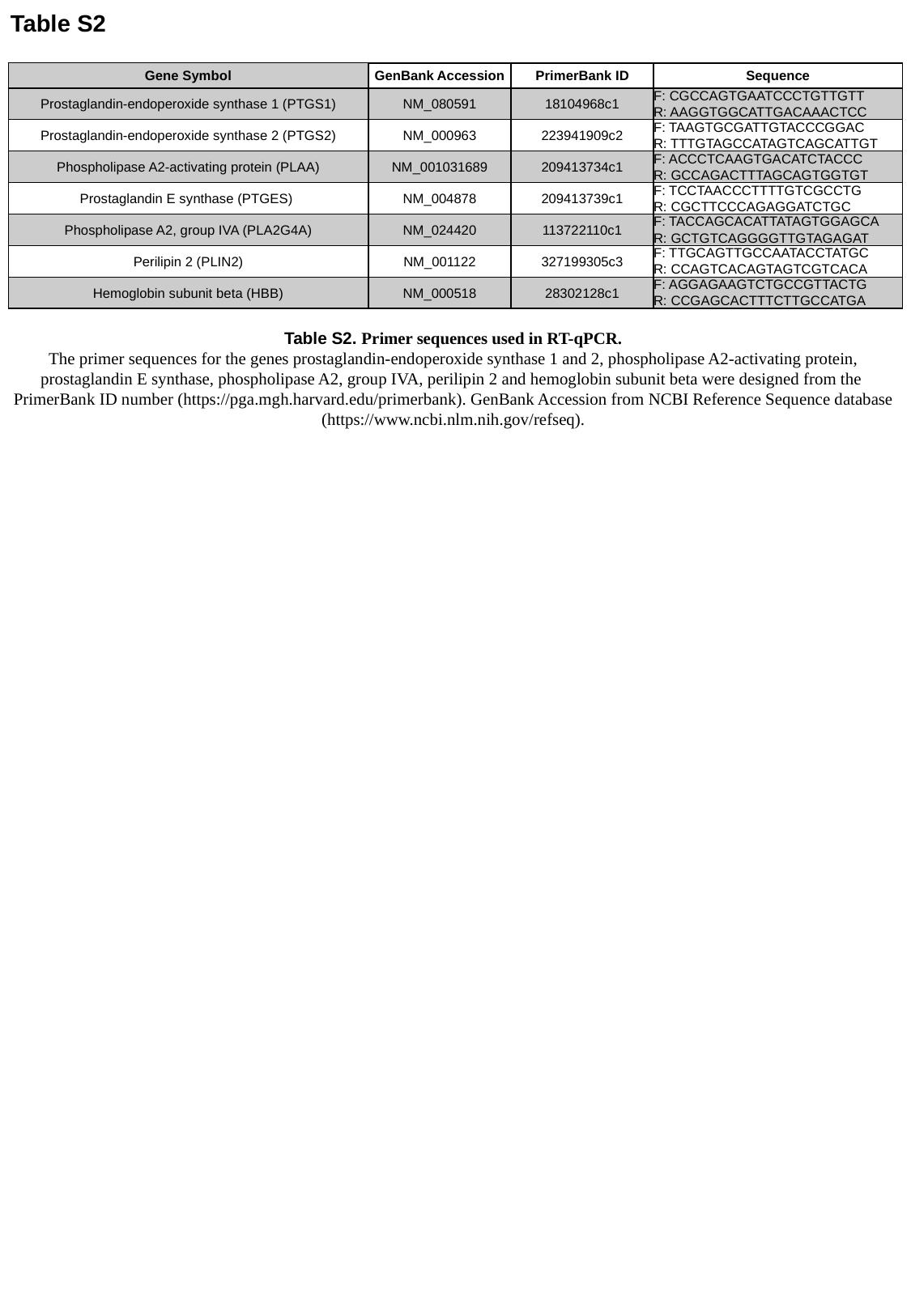

Table S2
| Gene Symbol | GenBank Accession | PrimerBank ID | Sequence |
| --- | --- | --- | --- |
| Prostaglandin-endoperoxide synthase 1 (PTGS1) | NM\_080591 | 18104968c1 | F: CGCCAGTGAATCCCTGTTGTT R: AAGGTGGCATTGACAAACTCC |
| Prostaglandin-endoperoxide synthase 2 (PTGS2) | NM\_000963 | 223941909c2 | F: TAAGTGCGATTGTACCCGGAC R: TTTGTAGCCATAGTCAGCATTGT |
| Phospholipase A2-activating protein (PLAA) | NM\_001031689 | 209413734c1 | F: ACCCTCAAGTGACATCTACCC R: GCCAGACTTTAGCAGTGGTGT |
| Prostaglandin E synthase (PTGES) | NM\_004878 | 209413739c1 | F: TCCTAACCCTTTTGTCGCCTG R: CGCTTCCCAGAGGATCTGC |
| Phospholipase A2, group IVA (PLA2G4A) | NM\_024420 | 113722110c1 | F: TACCAGCACATTATAGTGGAGCA R: GCTGTCAGGGGTTGTAGAGAT |
| Perilipin 2 (PLIN2) | NM\_001122 | 327199305c3 | F: TTGCAGTTGCCAATACCTATGC R: CCAGTCACAGTAGTCGTCACA |
| Hemoglobin subunit beta (HBB) | NM\_000518 | 28302128c1 | F: AGGAGAAGTCTGCCGTTACTG R: CCGAGCACTTTCTTGCCATGA |
Table S2. Primer sequences used in RT-qPCR.
The primer sequences for the genes prostaglandin-endoperoxide synthase 1 and 2, phospholipase A2-activating protein, prostaglandin E synthase, phospholipase A2, group IVA, perilipin 2 and hemoglobin subunit beta were designed from the PrimerBank ID number (https://pga.mgh.harvard.edu/primerbank). GenBank Accession from NCBI Reference Sequence database (https://www.ncbi.nlm.nih.gov/refseq).

## Slide 4
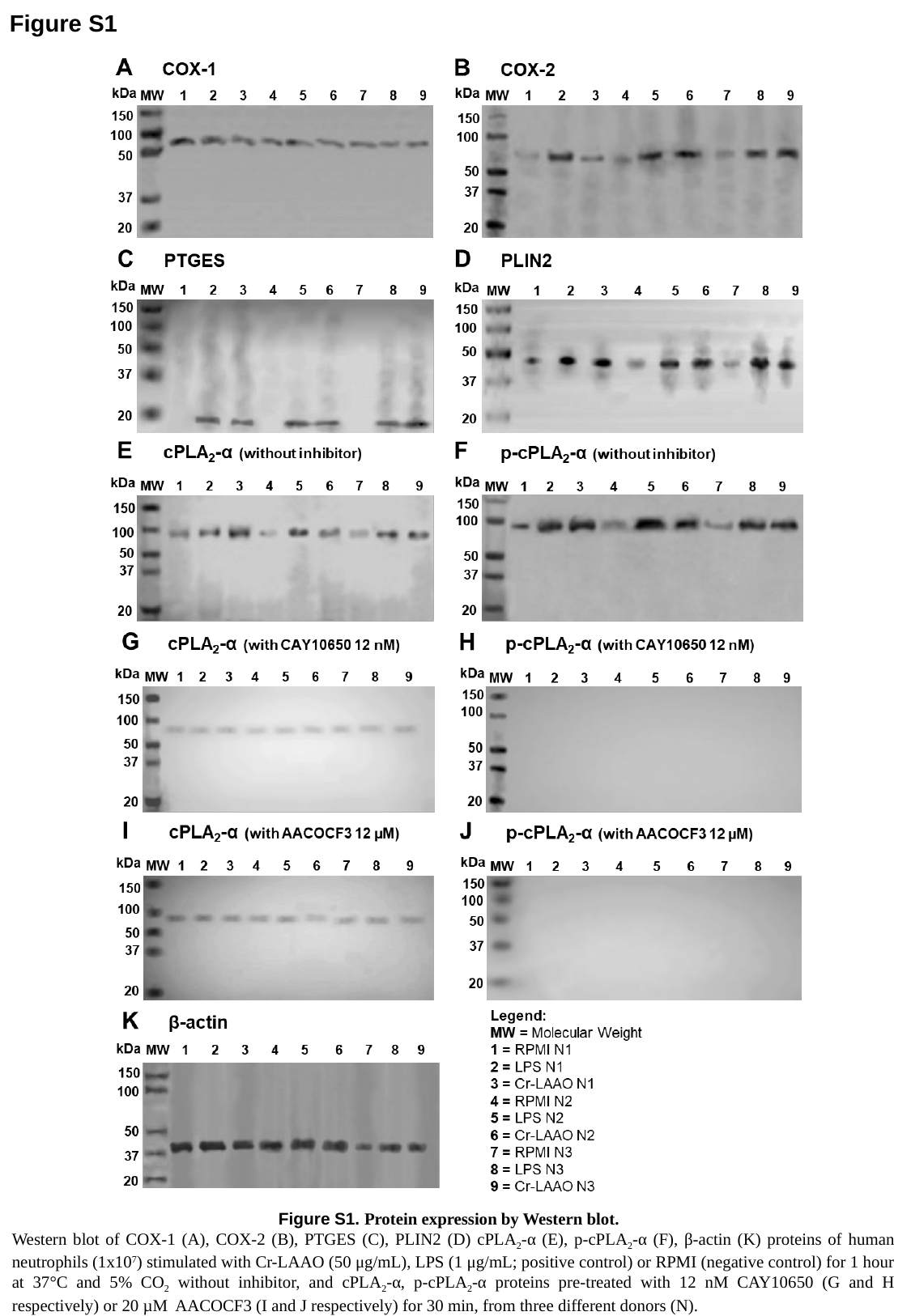

Figure S1
Figure S1. Protein expression by Western blot.
Western blot of COX-1 (A), COX-2 (B), PTGES (C), PLIN2 (D) cPLA2-α (E), p-cPLA2-α (F), β-actin (K) proteins of human neutrophils (1x10^7^) stimulated with Cr-LAAO (50 μg/mL), LPS (1 μg/mL; positive control) or RPMI (negative control) for 1 hour at 37°C and 5% CO2 without inhibitor, and cPLA2-α, p-cPLA2-α proteins pre-treated with 12 nM CAY10650 (G and H respectively) or 20 µM AACOCF3 (I and J respectively) for 30 min, from three different donors (N).
